# When Two Is Better than One: The Buddy-wire Technique in Flow-diversion Procedures

**DOI:** 10.1007/s00062-021-01053-x

**Published:** 2021-07-08

**Authors:** Tomas Dobrocky, Hubert Lee, Patrick Nicholson, Ronit Agid, Jeremy Lynch, Saravana Kumar Swaminathan, Timo Krings, Ivan Radovanovic, Vitor Mendes Pereira

**Affiliations:** 1 Division of Neuroradiology, Joint Department of Medical Imaging, Toronto Western Hospital, Toronto, Ontario Canada; 2 University Institute of Diagnostic and Interventional Neuroradiology, Inselspital, Bern University Hospital, University of Bern, Bern, Switzerland; 3 Division of Neurosurgery, Toronto Western Hospital, 399 Bathurst Street, M5T 2S8 Toronto, Ontario Canada

**Keywords:** Buddy-wire, Microcatheter navigation, Flow diversion, Intracranial aneurysm, Interventional neuroradiology

## Abstract

**Background:**

Delivery of most flow diverters (FD) requires larger, and thus stiffer microcatheters (0.021–0.027in.) which can pose challenges to intracranial navigation. The concomitant use of two microwires within one microcatheter, also known as the buddy-wire technique, may be helpful for navigation and support in challenging situations.

**Methods:**

We analyzed all flow diverter procedures in our prospectively collected database. We recorded all patient-related, anatomical and procedural information. We performed univariate statistics and technical descriptions.

**Results:**

In total, 208 consecutive patients treated with a FD at our institution between July 2014 and August 2020 were retrospectively analyzed. In 17 patients the buddy-wire technique was used (mean age 63 years, range 31–87 years: 16 female). Aneurysms were located at the petrous, cavernous, supraophthalmic internal carotid artery, and a proximal M2 branch in 2, 7, 7 and 1 patient(s), respectively. In all cases a 0.027in. microcatheter was used for device deployment. In 14 patients with a wide-necked aneurysm the buddy-wire provided additional support to advance the microcatheter and mitigated the ledge between the aneurysm neck and the parent artery or a side branch. In two giant cavernous aneurysms treated with telescoping FDs, the buddy-wire was used to re-enter the proximal end of the foreshortened FD.

**Conclusion:**

The buddy-wire is a useful technique in FD procedures to prevent herniation of the microcatheter into the aneurysm sack, in wide-necked aneurysms to mitigate the ledge effect between the aneurysm neck and the parent artery where the microcatheter tip may get stuck, or to enable re-entry into a foreshortened FD.

## Introduction

Endovascular treatment is revolutionizing intracranial aneurysm (IA) management and continues to expand with technological advances, such as flow diverting (FD) stents. The FD promote flow reduction and subsequent aneurysmal thrombosis in addition to vessel remodeling [1]. This has enabled occlusion of large wide-necked and fusiform aneurysms that were previously deemed difficult to treat or required deconstructive treatment (parent vessel occlusion) [2–6].

However, FD placement can be technically challenging. Many factors may influence the level of difficulty including microcatheter navigation and stability of the system. As compared to microcatheters typically used for coiling or for deployment of remodeling stents, microcatheters needed to accommodate FD have a larger inner diameter (0.021–0.027in.). Despite adequate proximal guide catheter support, microcatheter navigation in tortuous anatomy may be challenging or in certain aneurysm morphologies even impossible. Controlled navigation of neurovascular devices during the procedures is crucial as increasing build-up of forward load and uncontrolled release thereof may result in severe complications. Techniques previously proposed in the literature to overcome the limitation of navigating large bore microcatheters include exchange maneuvers, navigation of large distal access catheters to distal locations or the distal anchoring technique with stent-retriever stents or a balloon [7].

We herein report our experience with the buddy-wire technique by exploiting the additional luminal space of a 0.027in. microcatheter with a second or buddy microwire introduced in parallel to the commonly used 0.014in. microwire to facilitate intracranial navigation in complex IA treatment with FDs.

## Material and Methods

We performed a retrospective review of a prospectively collected database including all patients who underwent flow-diverter treatment at the Toronto Western Hospital, Toronto, from July 2014 to August 2020. The study was approved by our institutional ethics board (Nr. 16-6333), the need for a consent was waived due to the retrospective nature of the study.

All procedures performed in the studies involving human participants were in accordance with the ethical standards of the institutional and/or national research committee and with the 1964 Helsinki Declaration and its later amendments or comparable ethical standards.

The following inclusion criteria were applied: (1) endovascular treatment of a ruptured or unruptured intracranial aneurysm with a FD, employing (2) a 0.027in. microcatheter for deployment (3) insertion of a 0.010in. buddy-wire within the same microcatheter in parallel to a 0.014in. microwire at any time during the procedure.

The exclusion criteria were as follows: navigation of the appropriate microcatheter with a single microwire, or the use of microcatheter < 0.027in.

### Procedure

**Fig. 1:**
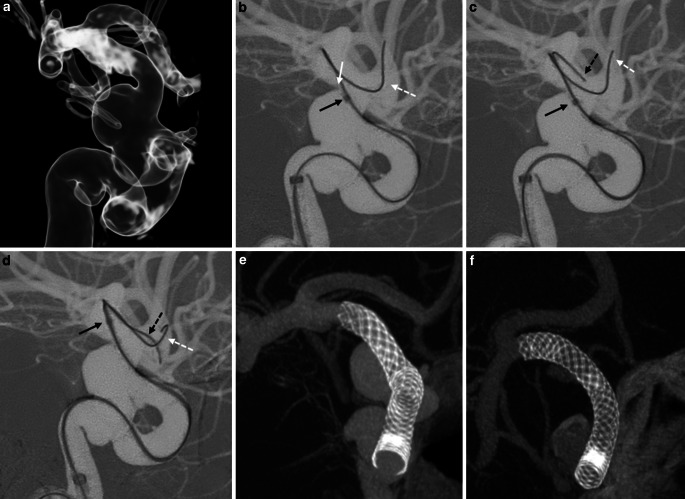
Female patient with an incidental right supraophthalmic internal carotid artery aneurysm. **a** Planning 3D-rotational angiogram demonstrated the wide-necked aneurysm on the posterior ICA wall with an acute angle between the aneurysm neck and the parent artery creating a ledge. **b** A Synchro 0.014in. microwire (*white dashed arrow*) was navigated across the aneurysm; however, due to the off-set between the 0.027in. microcatheter and the microwire the tip of the microcatheter (*black arrow*) was stuck on the ledge (*white arrow*) between the aneurysm neck and the parent artery preventing to be advanced distally. **c**,**d** An X‑pedion 0.010in. (Medtronic, Minneapolis, MN, USA) microwire (*black dashed arrow*) was then introduced into the Phenom 0.027in. parallel to the Synchro 0.014in. and provided the necessary support for the microcatheter to be advanced past the aneurysm and positioned distally. **e**,**f** A 4.75 × 18 mm Pipeline Flex embolization device (Medtronic, Minneapolis, MN, USA) was subsequently deployed. The cone beam CT demonstrated a good wall apposition

**Fig. 2:**
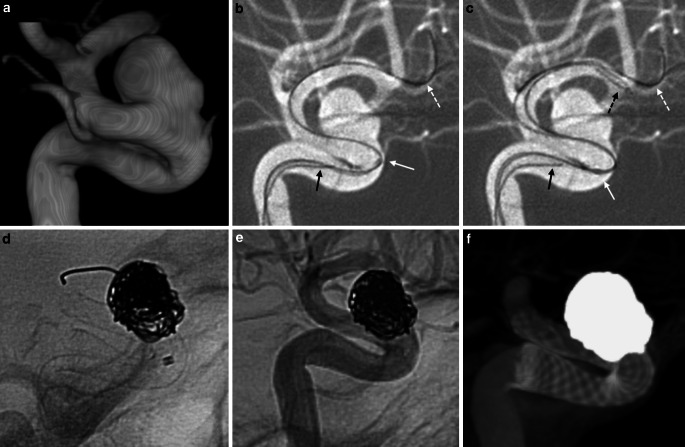
Female patient with a family history of aneurysmal subarachnoid hemorrhage and incidental left paraophthalmic internal carotid artery. **a** 3D angiogram demonstrates the wide-necked aneurysm just distal to the origin of the ophthalmic artery. **b** Attempts to navigate a Marksman microcatheter over a Synchro 0.014in. microwire positioned in the left M2 were unsuccessful with the catheter tip being caught on the ophthalmic artery origin (*white arrow*). Note, a second microcatheter with a wire (*black arrow*) is being advanced into the aneurysm which was later used for coiling. **c** Adding an X‑pedion 0.010in microwire (*black dashed arrow*) within the Marksman microcatheter, which was advanced similarly into the left M2, provided the necessary support for the microcatheter to traverse across the ophthalmic segment for distal positioning. **d**–**f** A 4.75 × 18 mm Pipeline Flex embolization device was subsequently deployed and the aneurysm was coiled

With the patient under general anesthesia, femoral access was obtained with an 8‑Fr sheath inserted by the Seldinger technique. Patients were anticoagulated with an i.v. bolus of 1000 IU heparin/10 kg body weight, followed by 1000 IU hourly thereafter. Digital subtraction and 3D flat panel angiography of the target vessel were acquired for planning. Next, a 6-French 90 cm guiding catheter (NeuronMAX, Penumbra, Alameda, CA, USA) was advanced into the proximal cervical internal carotid artery (ICA) or the first segment of the vertebral artery. A 0.027in. microcatheter (i.e. 150 cm Marksman or Phenom 27, ev3/Medtronic, Minneapolis, MN, USA, or Excelsior XT-27, Stryker Neurovascular, Fremont, CA, USA) was advanced over a microwire (Synchro‑0.014in, Stryker) past the target aneurysm. In cases of proximal vessel tortuosity an intermediate catheter was used in a triaxial fashion for additional support during subsequent FD deployment. In the case of challenging anatomy and wide-necked aneurysms, the microcatheter tip sometimes became stuck at the ledge between the aneurysm and the parent artery (Fig. 1) or at the origin of a side branch (i.e. the ophthalmic artery) (Fig. 2), preventing distal navigation of the catheter. In this scenario, a 0.010in. microwire was introduced into the same microcatheter alongside the 0.014in. microwire. This generally provided more support and reduced the offset between the microcatheter and the 0.014in. microwire allowing for advancement of the microcatheter over both wires past the ledge of the aneurysm or the ophthalmic artery.

After appropriate positioning of the microcatheter tip distal to the aneurysm both wires were removed. Next, the FD was advanced and deployed under fluoroscopic control covering the aneurysm neck. After deployment the FD was reaccessed by advancing the microcatheter inside the deployed FD over the delivery wire. The microcatheter was then used to oppose the device along the outer curvatures for better device opening and wall apposition (massaging technique). Next, a 2D run and a dedicated 3D flat panel cone beam CT were performed to confirm good wall apposition of the device [8]. In case of poor wall apposition a balloon angioplasty was performed using the Scepter (Microvention, Tustin, CA, USA) or Eclipse (Balt, Montmorency, France).

In patients with giant aneurysms requiring more than one FD to bridge the aneurysm sack, re-entering the proximal end of the FD proximally within the aneurysm fundus may pose a challenge. In these patients a buddy-wire was used to re-enter the FD and provide additional support for the microcatheter to be advanced distally.

## Results

In total, 208 consecutive patients treated at our institution between July 2014 and August 2020 with a flow-diverter were reviewed. Of these, 17 patients (mean age 63, range 31–87: 16 female) in whom a buddy-wire technique was used during the procedure were included. The locations of the target aneurysm included the petrous, cavernous, and supraophthalmic segments of the ICA in 2, 7, and 7 patients, respectively. In one patient a proximal M2 branch aneurysm was treated. In total, 9 of the treated aneurysms were considered giant (> 25 mm). Of the patients nine presented incidentally, six with ophthalmoplegia, one with retro-orbital pain, one with recurrence of previously ruptured aneurysm which has been initially coiled. Additional coiling of the target aneurysm was performed in six patients. In 13 patients a single FD was deployed, in 4 patients multiple FD were deployed in a telescoping fashion (range 2–4 FDs).

In 5 patients the buddy-wire used was a Transend 0.010in. (Stryker Neurovascular, Fremont, CA, USA) and in 12 patients the Xpedion-10 (Medtronic). XT-27 (Stryker), Phenom 27 (Medtronic), and Marksman (Medtronic) microcatheters were utilized in 6, 5 and 6 cases, respectively.

In 13 patients with wide-necked aneurysms located along the outer curvature of the ICA the buddy-wire was used to provide additional support to advance the microcatheter distal to the aneurysm. In two patients with giant cavernous aneurysms treated with multiple FDs deployed in a telescoping fashion, the buddy-wire was used to re-enter the proximal end of the FD which was not bridging the aneurysms or had foreshortened after balloon angioplasty. In one patient with a proximal M2 segment aneurysm the buddy-wire was used to enable advancement of the microcatheter past the MCA bifurcation which initially prevented distal navigation due to a ledge effect at the bifurcation.

We hereby demonstrate three cases to illustrate the various scenarios in which we utilized the buddy-wire technique:

### Scenario 1: Distal Access Across Giant/Wide-necked Aneurysms

A female patient with a large, wide-necked incidental right supraophthalmic ICA aneurysm. During the intervention a Synchro 0.014in. microwire was navigated past the aneurysm; however, the catheter slightly herniated into the aneurysm sack and due to the off-set between the 0.027in. microcatheter and the 0.014in. microwire, the catheter tip got caught on the ledge between the aneurysm neck and the parent artery, preventing distal navigation. A 0.010in. microwire was inserted into the 0.027in. microcatheter alongside the 0.014in. microwire, and navigated past the aneurysm, along the first microwire into the M1 segment. The additional support of the buddy-wire helped deflect the tip of the microcatheter away from the aneurysm ledge and we were able to advance the microcatheter across the aneurysm to a satisfactory position for stent deployment (see Fig. 1).

### Scenario 2: Navigation Past the Ophthalmic Artery

A female patient with a family history of aneurysmal subarachnoid hemorrhage and incidental, wide-necked left paraophthalmic ICA aneurysm. Attempts to navigate a Marksman microcatheter over a Synchro 0.014in. microwire failed due to the catheter tip becoming caught on the ophthalmic artery origin. Addition of an X‑pedion 0.010in. microwire in the Marksman microcatheter, provided the necessary support for the microcatheter to traverse the ophthalmic segment for distal positioning. (see Fig. 2).

### Scenario 3: Re-entry into a Foreshortened FD

**Fig. 3:**
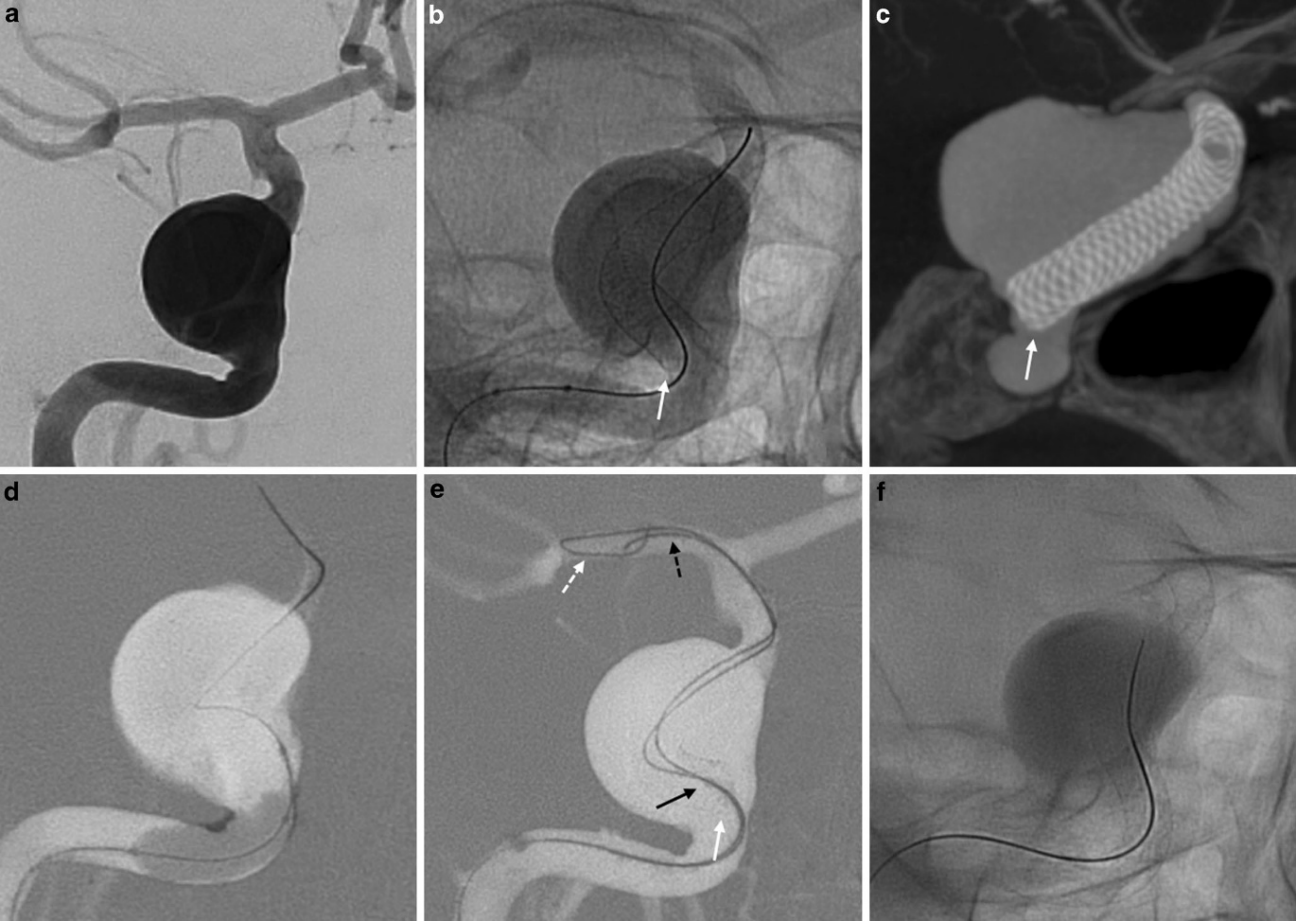
Female patient presenting with right-sided ophthalmoplegia. The CT angiogram (CTA, not shown), and later an intraprocedural prestenting angiogram (**a**), demonstrated a large ipsilateral cavernous carotid aneurysm. **b** Treatment was pursued using a 4 mm × 50 mm Stryker Surpass Streamline flow diverting stent delivered through a Stryker XT-27 microcatheter. The proximal end of the FD (*white arrow*) landing a little too short. **c** Cone beam CT demonstrated poor wall apposition of the proximal end of the FD (*white arrow*). (**d**) Angioplasty of the proximal FD was performed to improve wall apposition. **e** This resulted in foreshortening and distal migration of the proximal end of the FD (*white arrow*) upon balloon removal, with prolapse of the proximal stent into the aneurysm. Access into and through the stent lumen was regained using the buddy-wire technique with a Synchro 0.014in microwire (*white dashed arrow*) and X‑pedion 0.010in. guidewire (*black dashed arrow*) allowing distal navigation of the XT-27 microcatheter (tip of the catheter, *black arrow*). **f** A second Surpass Streamline FD was deployed telescopically covering the proximal end of the aneurysm and improving wall apposition of the first FD

A female patient presenting with right ophthalmoplegia. The CT angiogram demonstrated a giant ipsilateral cavernous carotid aneurysm. Treatment was pursued using a 4 mm × 50 mm Stryker Surpass Streamline flow diverting stent delivered through a Stryker XT-27 microcatheter. Angioplasty of the stent was performed to improve wall apposition resulting in foreshortening of the proximal end of the FD into the aneurysm sac. To regain access an initial attempt was made with the 0.014in. microwire; however, this did not provide sufficient support to advance the microcatheter back into the stent. The 0.014in. microwire was used to anchor the proximal end of the stent by hooking its tines. A 0.010in. microwire with a tight J‑shaped curve was then introduced through the same 0.027in. microcatheter and used to select the lumen of the stabilized stent. After traversing the stent and gaining distal access with the 0.010in. microwire, the second microwire was directed along the same path as the initial microwire and provided sufficient support to progressively advance the microcatheter distally (see Fig. 3).

## Discussion

**Fig. 4:**
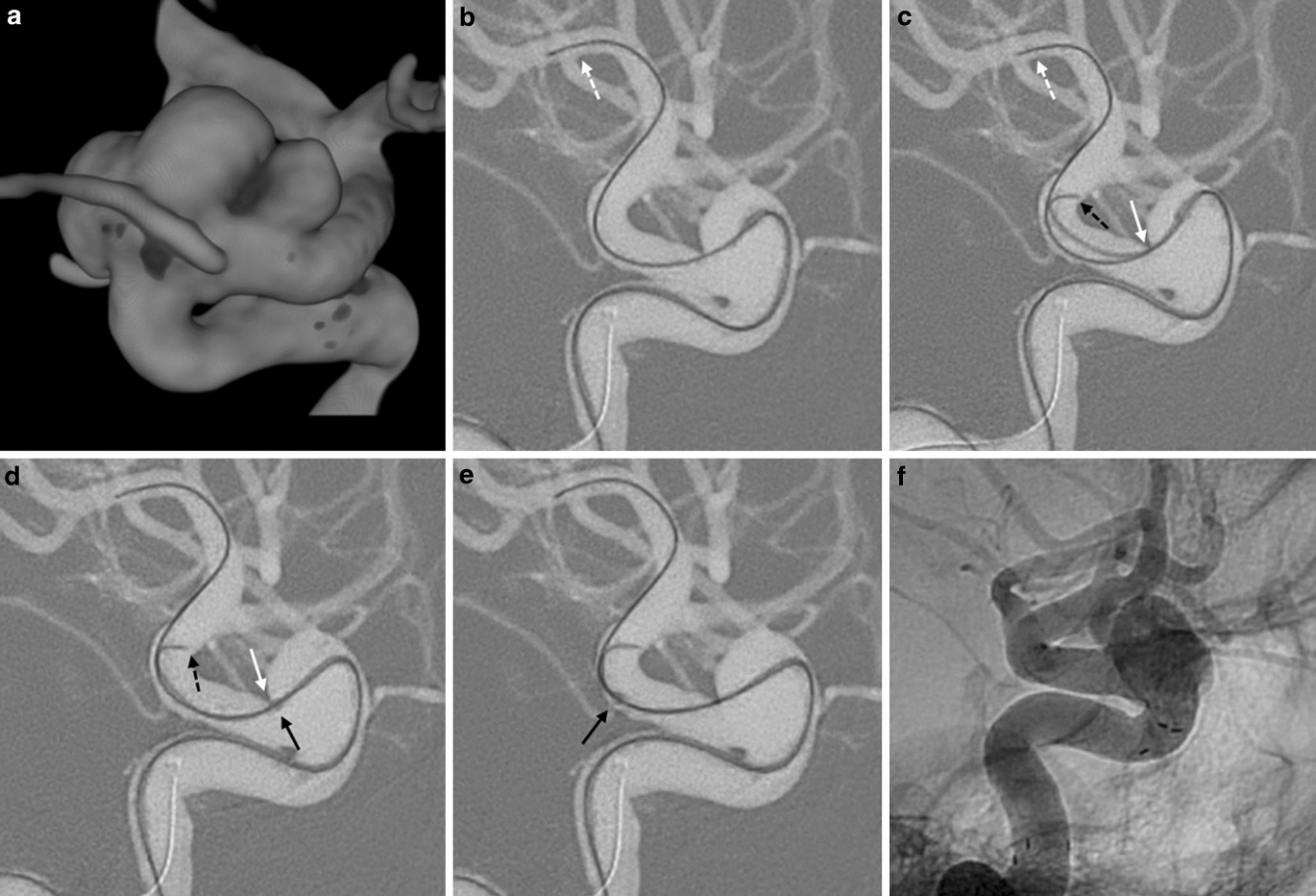
Female patient with an incidental right paraophthalmic ICA aneurysm. **a** Periinterventional planning 3D-rotational angiogram demonstrated the wide-necked, trilobulated morphology of the aneurysm. **b** A Synchro 0.014in. microwire (*white dashed arrow*) was navigated across the aneurysm. **c**–**e** Due to the lacking support of a single microwire and stiffness of the XT-27 microcatheter the catheter bulged into the aneurysm sac, and due to the off-set between the microwire and the tip of the microcatheter (*black arrow*) was stuck on the ledge (*white arrow*) between the aneurysm neck and the parent artery preventing to be advanced distally. A Transend 0.010in. microwire (*black dashed arrow*) was then introduced into the XT-27 in parallel to the Synchro 0.014in providing more support for the microcatheter to be advanced past the aneurysm and positioned distally. A Surpass Evolve (Stryker Neurovascular, Fremont, CA, USA) was subsequently deployed. The cone beam CT (not shown) demonstrated some fish-mouthing at the proximal end of the FD. **f** A Neuroform Atlas stent was then deployed to improve wall apposition in the proximal part of the FD

Large caliber microcatheters are necessary for the deployment of most current flow diverting stents. Compared to smaller catheters used for aneurysm coiling, these larger microcatheters result in reduced flexibility, which can impact distal vessel navigation. We have demonstrated that in approximately 10% of FD cases, there are situations in which despite good proximal support, the appropriate positioning of the 0.027in. microcatheter distal to the aneurysm or access into an already deployed FD is not possible. In this technical report we describe a potential solution—the buddy wire technique—by adding a 0.010in. microwire alongside a 0.014in. guidewire within a 0.027in. microcatheter. Both microwires together mimic the support of a larger caliber microwire improving trackability of the microcatheter in tortuous anatomy. This becomes necessary for crossing wide-necked aneurysms situated at or near acute angulations in the vessel where the vector of force points into the aneurysm fundus potentiating herniation of the microcatheter (Fig. 1 and 4). The added stiffness of two microwires may also straighten the artery to be selectively catheterized producing a more favorable trajectory.

In addition, the disparity in diameter between the 0.014in. microwire and 0.027in. microcatheter may create a ledge at the tip of the device which can act as a barrier to distal access. The microcatheter tip can either become caught on the origin of arterial branches, such as the ophthalmic artery or on the neck of the aneurysm. The addition of the buddy-wire reduces the space between the wire and catheter ledge evening out the ratio between their diameters. A similar effect can be achieved with the addition of a smaller microcatheter introduced in a coaxial manner, which has also been utilized to advance distal aspiration catheters for endovascular thrombectomy in stroke [9]. The buddy-wire offers a cost-effective, easy to implement alternative that eliminates the ledge effect while reducing the amount of catheter dead space to improve pushability of the microcatheter through challenging vascular anatomy associated with wide-necked aneurysms.

Owing to its smaller diameter, 0.010in. microwires are softer and allow easier navigation without creating too much push back. This is especially advantageous when re-entering the lumen of a deployed flow diverting stent that has become loose or has foreshortened in a giant aneurysm fundus requiring telescoping deployment of additional FD. It is not routinely used for aneurysm treatment as it intrinsically provides poor support for microcatheter tracking; however, as an adjunct to a more commonly implemented 0.014in. microwire, it can secure the access and provide the support required to achieve distal positioning to the aneurysm for stent deployment. This will become particularly pertinent as more distal aneurysms beyond the circle of Willis are increasingly considered for flow diversion.

The buddy-wire technique is an alternative to the previously reported anchoring technique in situations of microcatheter looping in the sac of a large or giant aneurysm. The later constitutes a primary catheterization of the distal vessel segment with a more flexible microcatheter (i.e. SL-10). The microwire is then removed and a low-profile stent retriever or a balloon is deployed [10]. The shear force against the endothelial wall generated by the SR struts or an inflated balloon is used as an anchor to slowly unravel the microcatheter from within the aneurysm dome. Whilst this technique spares exchange maneuvers using long wires (length 300 cm), which are particularly prone to vessel injury due to distal wire perforation, it involves some challenges. This is mainly due to the short length of the SR pusher wire which is too short for conventional exchange. Furthermore, additional use of a primary catheter, stent or balloon results in higher procedure costs.

Use of the buddy-wire has been described in the interventional cardiology literature as a means of increasing support to cross highly calcified and stenotic lesions, during balloon angioplasty, or navigating tortuous anatomy [11, 12]. These techniques positioned the guidewire parallel, but outside the main microcatheter improving stability of the guiding catheter, increasing friction between the balloon and neighboring tissues or favorably altering the vessel anatomy. This technique has been reported in neurointerventions for vertebral artery origin stenting, or coil embolization of posterior circulation aneurysms in patients with proximal vessel tortuosity. In these cases stability of the guiding catheter was achieved by adding a buddy-wire placed in the subclavian artery [13–15]. Some case reports have demonstrated the application of the buddy-wire technique during coiling of a wide-necked basilar tip aneurysm [16], to facilitate Y‑stenting in a middle cerebral artery bifurcation aneurysm [17], navigation of a large microcatheter for treatment of ACOM aneurysm with a WEB device [18], or improving support during treatment of a dissecting cavernous ICA aneurysm [19].

Our study has some limitations inherent to its monocentric and retrospective design. However, it reports a consecutive series of patients treated with a FD and identifies scenarios in which the buddy wire technique may be useful. This manuscript describes the potential applications of the buddy-wire technique and does not compare its success to other alternative methods.

## Conclusion

Use of larger and thus stiffer microcatheters required for flow diverter treatment of intracranial aneurysms may lead to difficulty negotiating tortuous anatomy. Insertion of a second buddy-wire within the same microcatheter augments support, favorably alters the vessel course, removes space between the wire and catheter tip (ledge effect), and may help to regain access to a FD that has foreshortened within the aneurysm fundus. The buddy-wire technique serves as an effective tool to complement the neuroendovascular armamentarium.

## Abbreviations

CTA: Computed tomography angiogram
FD: Flow diverter
IA: Intracranial aneurysm
ICA: Internal carotid artery
